# Mesoamerican Nephropathy and Kidney Disease Progression: A Case Series of Individuals With Kidney Biopsies From Nicaragua and El Salvador

**DOI:** 10.1016/j.xkme.2021.04.016

**Published:** 2021-07-05

**Authors:** Julia Wijkström, Marvin Gonzalez-Quiroz, Ricardo Leiva, Zulma Cruz Trujillo, Carl-Gustaf Elinder, Annika Wernerson

**Affiliations:** 1 Division of Renal Medicine, Department of Clinical Science, Intervention and Technology (CLINTEC), Karolinska Institutet, Stockholm, Sweden; 2 Department of Renal Medicine, Karolinska University Hospital, Stockholm, Sweden; 3 Research Centre on Health, Work and Environment (CISTA), National Autonomous University of Nicaragua, León (UNAN-León), León, Nicaragua; 4 Department of Renal Medicine, University College London, London, United Kingdom; 5 Department of Nephrology, Hospital Nacional Rosales, San Salvador, El Salvador

To the Editor:

Mesoamerican nephropathy (MeN) is a chronic kidney disease affecting rural populations in Central America, especially sugarcane workers.^1^ Prospective studies and data regarding the natural history of the disease are scarce. We have previously described kidney morphology and biochemical characteristics of MeN in 2 case series,^2^^,^^3^ including a 1-to-2-year-long follow-up where mean estimated glomerular filtration rate (eGFR) decline was 4.4 mL/min/1.73 m^2^ per year.^3^ Another 2-year-long prospective study of young rural men with chronic kidney disease in Nicaragua without kidney biopsies reported an average eGFR decline of 3.8 mL/min/1.73 m^2^.^4^ We now report eGFR trends in individuals with MeN with kidney biopsies over a period of 5-7 years.

Participants from our previous kidney biopsy studies in El Salvador and Nicaragua^2^^,^^3^ were invited to participate in this long-term follow-up. Blood and urine samples were collected and the subset of participants from Nicaragua also completed a questionnaire regarding work history. eGFR was calculated using the CKD-EPI equation.^5^ Samples were analyzed at the same local laboratory as in the baseline study. Kidney biopsy findings at baseline are described in previous publications.^2^^,^^3^ In hypothesis-generating analyses, associations between baseline biopsy findings and progression rate were calculated using linear regression for continuous variables and 1-way analysis of variance for categorical variables (GraphPad). Ethics committees at Hospital Nacional Rosales, El Salvador (ACTA EXP. No 11/2016), the National Autonomous University of Nicaragua, Nicaragua (ACTA No 31 and 83), and Stockholm, Sweden (Dnr 2015/849-32) approved the study. New written informed consent was collected from Nicaragua participants. No new informed consent was required from the ethics committee in El Salvador (written informed consent was obtained at baseline kidney biopsy study).

There were 26 patients included after a mean of 5.2 years from biopsy. From the El Salvador study 7 of 8 participants were included; 1 was lost to follow-up. One of the included participants died during follow-up (last available eGFR was used for calculations). From the Nicaragua study all 19 participants were included.

**Figure 1:**
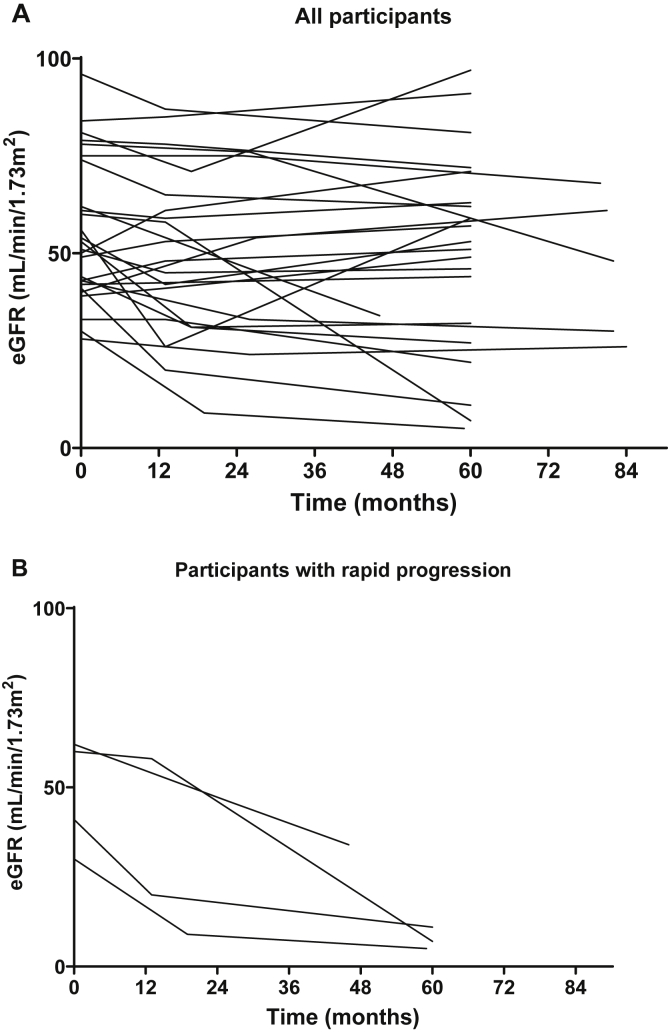
Estimated glomerular filtration rate (eGFR) development during 5-to-7-year follow-up of Mesoamerican nephropathy (MeN) patients with biopsy-proven MeN. (A) All 26 participants. (B) Four patients had a rapid progression (defined as eGFR decline >5 mL/min/1.73 m^2^ per year).

The change in eGFR during follow-up is presented in Table 1 and Fig 1. Individual biochemical, morphological, and questionnaire data are presented in Tables S1, S2, and S3, respectively. Mean change in eGFR was -1.7 ± 4.1 mL/min/1.73 m^2^ per year. Half of the participants had a decline in eGFR greater than 1.0 mL/min/1.73 m^2^ per year, while 4 participants had a rapid progression, defined as a ΔeGFR of -5 mL/min/1.73 m^2^ per year or more.^6^ Serum sodium, potassium, and magnesium levels at follow-up were similar to baseline values, with hypokalemia found in 6 of 26 patients and hypomagnesemia in 6 of 19 patients. Low serum levels of potassium and magnesium were found in patients both with and without progressive disease. The 2 patients with the most rapid progression had the highest levels of albuminuria at baseline, with urinary albumin-creatinine ratio of 788 and 809 mg/g, respectively (89 and 91 mg/mmol), although the 2 additional participants with rapid progression did not have significant albuminuria (urinary albumin-creatinine ratio 2 and 29 mg/g [0.2 and 3.3 mg/mmol]). Blood pressure at follow-up was <140/90 in all but 6 patients. One of the patients with rapid progression had very high blood pressure (216/122) that could have affected kidney function (Table S1).

**Table 1:** Follow-up Data of 2 Patient Case Series With Mesoamerican Nephropathy 1-2 Years (F1) and 4-7 Years (F2) After Kidney Biopsy Study

	El Salvador, N = 7	Nicaragua, N = 19	All Participants, N = 26
Age at T0, y	43 ± 13 (22-57)	33 ± 8 (24-54)	36 ± 10
Follow**-**up time F1, y	2.1 ± 0.2 (1.6-2.3)	1.1 ± 0.1 (1.1-1.4)	1.4 ± 0.5
Follow**-**up time F2, years	5.6 ± 2.1 (3.8-7.0)	5.0 ± 0	5.2 ± 1.1
eGFR T0, mL/min/1.73 m^2^	51 ± 27 (28-78)	57 ± 18 (33-96)	56 ± 18
eGFR F1, mL/min/1.73 m^2^	45 ± 25 (9-76)^a^	52 ± 20 (20-87)^b^	50 ± 20
eGFR F2, mL/min/1.73 m^2^	39 ± 22 (5-68)^c^	52 ± 25 (7-97)	49 ± 23^c^
Declining eGFR >1 mL/min/1.73 m^2^ per y	5 (71 %)	8 (42 %)	13 (50 %)
Declining eGFR >5 mL/min/1.73 m^2^ per y	2 (29 %)	2 (11 %)	4 of 26 (15 %)
ΔeGFR per y	-3.6 ± 5.3 (-13.3 to 3.1)	-1.0 ± 3.5 (-10.6 to 4.2)	-1.7 ± 4.1 (-13.3 to 4.2)

Values are presented as mean ± SD (range) or count (percentage).

Abbreviations: T0, baseline; F1, first follow-up; F2, second follow-up; eGFR, estimated glomerular filtration rate; ΔeGFR, change in eGFR.

a One patient started hemodialysis; eGFR on last appointment before dialysis start used in calculation.

b N = 18.

c One patient was diseased at second follow-up; kidney function eGFR 5 mL/min/1.73 m^2^ was estimated for this individual.

The degree of interstitial fibrosis at baseline was associated with eGFR decline, while the percentage of global glomerulosclerosis showed a similar but nonsignificant association (Figs S1 and S2). Segmental sclerosis was more common in patients with rapid progression (50 % compared to 15 % in the whole study group). No associations were found between progression rate and tubular atrophy, interstitial inflammation, or glomerular size.

All Nicaragua participants had worked in sugarcane fields before kidney biopsy, while only 11 of 19 participants had performed agricultural work tasks during follow-up, mostly subsistence farming (Table S3). Only 1 participant still worked with sugarcane during follow-up. Among patients with an eGFR decline >1 mL/min/1.73 m^2^ per year, 50 % reported physically demanding work during the last 3 months (including sugarcane seed cutter, tricycle driver, and farmer) compared to 27 % in the patients with stable or improved eGFR (including farmer, tractor driver). Self-reported agrochemical exposure was found in 50 % of patients with eGFR decline >1 mL/min/1.73 m^2^ compared to 27 % in the patients with stable eGFR. None of the patients with rapid decline in eGFR reported agrochemical exposure.

In conclusion, this long-term follow-up of 26 patients with biopsy-confirmed MeN shows a mean eGFR decline of -1.7 mL/min/1.73 m^2^ per year, with 15 % of participants having a rapid progression, 35 % a decline in eGFR between -5 and -1 mL/min/1.73 m^2^ per year, and 50 % stable or improved eGFR. Individuals with moderate-to-severe interstitial fibrosis in biopsies had a higher risk of progressive disease, and segmental sclerosis and albuminuria were more common in patients with rapid progression and may indicate a more active disease.

Heat stress and physically strenuous work have been identified as risk factors for MeN,^1^ with field studies of sugarcane workers showing that the incidence of eGFR decline over a harvest season is most pronounced among those individuals with the highest work and heat load, cane cutters.^7^ Indeed, physically demanding jobs were somewhat more common in patients with declining eGFR in this study. However, most participants had stopped working at sugarcane plantations after being diagnosed with MeN, which probably induces a significant bias.

Many participants had a more substantial drop in eGFR at their initial follow-up, with less rapid decline or even improvement in eGFR thereafter (Fig 1A). One could speculate whether the lower progression rate in this long-term follow-up, -1.7 mL/min/1.73 m^2^ per year (compared to short-term -4.4 mL/min/1.73 m^2^ per year), is due to a change in work tasks after diagnosis. Of the 19 participants from Nicaragua, all but 1 stopped working with sugarcane, potentially improving the long-term progression rate in this study. Furthermore, the progression rate may have been influenced by other factors such as improved medical surveillance, electrolyte supplements, lifestyle changes, and improved volume intake. In fact, a recent intervention study showed that improving working conditions for cane cutters, providing “water, shade, and rest,” decreases the risk for eGFR decline after harvest.^8^

Limitations of the study include the small sample size, with only 4 patients having a rapid progression rate, limiting the conclusions that can be drawn regarding risk factors for progression.

In summary, this follow-up study indicates that the natural history of MeN is diverse, with rapid progression in some patients and more stable kidney function in others. The degree of interstitial fibrosis in kidney biopsies was connected to a more negative outcome, and segmental sclerosis and albuminuria was seen in half of the patients with rapid deteriorating kidney function. Our findings suggest that medical surveillance and avoiding physically strenuous jobs is likely beneficial for maintaining kidney function in patients with MeN.
