# Supplementary material for: Mesoamerican Nephropathy and Kidney Disease Progression: A Case Series of Individuals With Kidney Biopsies From Nicaragua and El Salvador
Source: Kidney Med. 2021 Jul 5;3(5):871–3. doi: 10.1016/j.xkme.2021.04.016 (PMC8515091; doi:10.1016/j.xkme.2021.04.016)
Supplement: Supplementary File (PDF) — Figures S1-S2; Tables S1-S3. [file mmc1.pdf]

Figure S1: Interstitial fibrosis at baseline and change in eGFR. The degree of interstitial fibrosis at the time of the biopsy is significantly correlated with a decline in eGFR. One-way ANOVA, $F(3,20)=6.397$, $p= 0.003$. Graph shows individual values and median. Semi-quantification of interstitial fibrosis in the cortical area defined as: 0= no fibrosis; 1= ≤25% fibrosis; 2= 26-50% fibrosis; 3> 50% fibrosis.

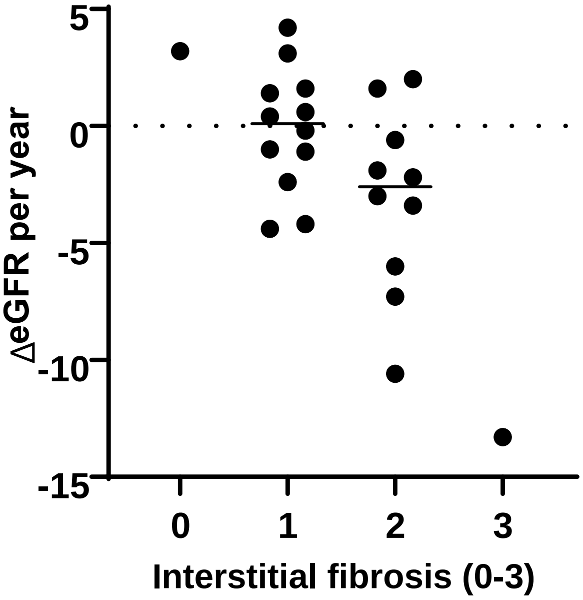

Figure S2: Percentage of glomerulosclerosis at baseline and change in eGFR. The percentage of glomerulosclerosis at the time of the biopsy shows a tendency, but non-significant association with a decline in eGFR. Linear regression, p=0.09.

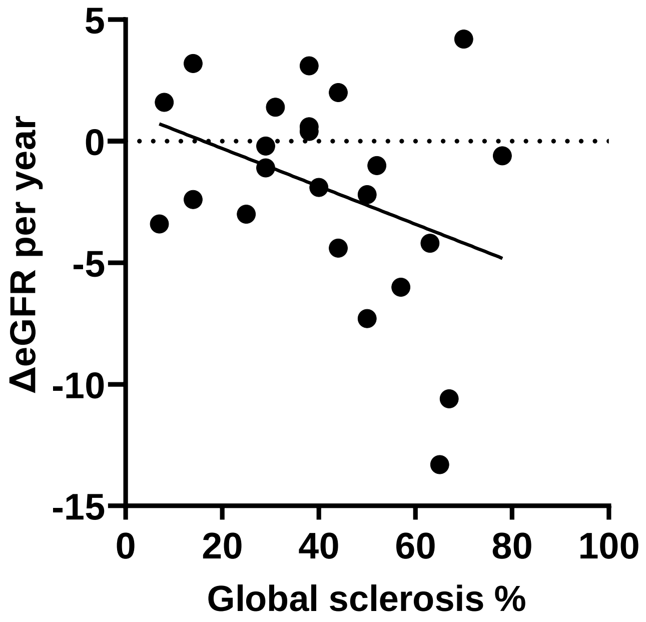

Table S1: Individual biochemical follow-up data of two patient case series with Mesoamerican Nephropathy from El Salvador and Nicaragua 4-7 years (F2) after kidney biopsy study.

**Pat no**	**eGFR T0**	**eGFR F2**	**Follow-up time T0-F2**	**ΔeGFR per year**	**S-Na T0**	**S-Na F2**	**S-K T0**	**S-K F2**	**S- Mg T0**	**S-Mg F2[a]**	**ACR T0**	**ACR F2**	**24h-U T0[b]**
**ES-1**	62	34	3.8	-7.3	142	139	3.9	3.4[c]	1.34[c]	-	2	-/[d]	-
**ES-2**	30	5	1.6	-13.3	139	139	5.3	3.4[c]	1.8	-	788	-/[d]	7.5
**ES-3**	40	61	6.8	3.1	137	137	4.1	3.6	2.02	-	6	-/[d]	-
**ES-4**	43	30	6.8	-1.9	140	138	3.4[c]	2.8[c]	1.26[c]	-	8	-/[d]	-
**ES-5**	78	48	6.8	-4.4	137	136	2.6[c]	2.4[c]	0.88[c]	-	7	-/[d]	-
**ES-6**	28	24	7.0	-0.6	141	139	4.1	3.8	1.58	-	12	-/[d]	-
**ES-7**	75	68	6.7	-1.1	139	137	3.6	3.3[c]	1.22[c]	-	17	-/[d]	2.5
**Nic-1**	53	32	5.0	-4.2	134	136	4.4	5.7	1.46	1.82	5	18	3.2
**Nic-2**	49	57	5.0	1.6	138	140	3.2[c]	4.3	1.26[c]	1.42	1	13	2.0
**Nic-3**	50	71	5.0	4.2	135	139	3.5	4.7	1.20[c]	1.04[c]	3	5	1.9

**Nic-4**	39	49	5.0	2.0	138	138	3.6	4.2	1.38[c]	1.46	4	31	2.5
**Nic-5**	44	27	5.0	-3.4	137	140	3.8	4.7	1.08[c]	1.08[c]	2	35	3.5
**Nic-6**	84	91	5.0	1.4	136	136	4.9	3.4[c]	1.56	1.78	1	10	3.0
**Nic-7**	54	53	5.0	-0.2	135	137	2.2[c]	4.8	1.50	1.48	317	18	5.3
**Nic-8**	56	59	5.0	0.6	136	141	4.7	5.1	1.46	1.40	8	30	1.6
**Nic-9**	74	62	5.0	-2.4	137	141	3.8	5.1	1.62	1.60	9	13	3.7
**Nic-10**	81	97	5.0	3.2	137	139	4.5	4.7	1.58	1.80	1	8	1.3
**Nic-11**	79	72	5.0	-1.4	137	139	3.7	4.3	1.58	1.76	2	7	3.7
**Nic-12**	33	22	5.0	-2.2	136	139	3.6	4.1	1.02[c]	0.98[c]	3	9	4.8
**Nic-13**	61	63	5.0	0.4	138	138	3.6	4.7	1.60	1.30[c]	2	30	3.2
**Nic-14**	42	44	5.0	0.4	137	139	3.1[c]	4.0	1.10[c]	1.04[c]	1	8	1.7
**Nic-15**	41	11	5.0	-6.0	133	137	4.3	3.9	1.02[c]	1.30[c]	29	14	2.1
**Nic-16**	43	51	5.0	1.6	139	139	3.7	4.4	1.64	1.66	2	60	2.8
**Nic-17**	51	46	5.0	-1.0	139	138	4.2	4.6	1.54	1.64	7	7	3.6
**Nic-18**	96	81	5.0	-3.0	136	136	3.8	4.5	1.62	1.50	13	6	6.7

**Nic-19**	60	7	5.0	-10.6	136	139	3.3[c]	5.0	1.50	2.46	809	143	2.2
**Mean**	**56**	**49**	**5.2**	**-1.7**	**137**	**138**	**3.8**	**4.2**	**1.42**	**1.50**	**79**	**25**	**3.3**
**SD**	**18**	**23**	**1.1**	**4.1**	**2**	**1**	**0.7**	**0.8**	**0.27**	**0.36**	**220**	**32**	**1.6**
**Unit**	ml/min/$1.73m^2$		years	ml/min/$1.73m^2$/year	mEq/L	mEq/L	mEq/L	mEq/L	mEq/L	mEq/L	mEq/L	mg/g	L

Abbreviations: T0, baseline; F2, second follow-up; Δ eGFR, change in eGFR; S-Na, serum sodium; S-K, serum potassium; ACR, urine albumin creatinine ratio; 24h-U, 24-hour urine; SD, standard deviation.

Reference values: S-Na, 137-145 mEq/L; S-K, 3.5-5.0 mEq/L; S-Mg, 1.40-1.90 mEq/L; UACR, <30 mg/g.

Conversion factors for units: ACR in mg/g to mg/mmol, ×0.113.

[a] S-Mg at follow-up not available in patients from El Salvador.

[b] 24-hour urine collection only available in two patients from El Salvador.

[c] Below refence value.

[d] Incomplete data.

Table S2: Baseline eGFR, progression rate, and kidney biopsy findings at baseline in two patient case series with Mesoamerican Nephropathy from El Salvador and Nicaragua.

Pat no	eGFR T0	ΔeGFR per year	Global scleros (%)[a]	Segm scleros (0-1)[b]	Interst fibrosis (0-3)[c]	Interst inflam (0-3)[c]	Tubular atrophy (0-3)[c]	Glom size (0-3)[d]	Wrinkl GBM/ Periglom fibrosis (0-1)[e]
**ES-1**	62	-7.3	50%	0	2	1	1	2	1
**ES-2**	30	-13.3	65%	1	3	2	2	3	1
**ES-3**	40	3.1	38%	0	1	1	1	1	1
**ES-4**	43	-1.9	40%	0	2	2	2	2	1
**ES-5**	78	-4.4	44%	0	1	1	1	1	1
**ES-6**	28	-0.6	78%	1	2	1	2	3	1
**ES-7**	75	-1.1	29%	0	1	0	1	1	1
**Nic-1**	53	-4.2	63%	0	1	1	1	3	1
**Nic-2**	49	1.6	n/a[f]	0	1	1	0	1	0

**Nic-3**	50	4.2	70%	0	1	1	1	2	1
**Nic-4**	39	2.0	44%	0	2	2	2	2	1
**Nic-5**	44	-3.4	7%	0	2	1	1	3	1
**Nic-6**	84	1.4	31%	0	1	1	1	2	1
**Nic-7**	54	-0.2	29%	1	1	1	1	3	1
**Nic-8**	56	0.6	38%	0	1	1	1	3	1
**Nic-9**	74	-2.4	14%	0	1	1	1	2	1
**Nic-10**	81	3.2	14%	0	0	0	0	2	0
**Nic-11**	79	-1.4	n/a[f]	-	-	-	-	-	-
**Nic-12**	33	-2.2	50%	0	2	2	1	2	1
**Nic-13**	61	0.4	38%	0	1	0	1	2	1
**Nic-14**	42	0.4	n/a[f]	-	-	-	-	-	-
**Nic-15**	41	-6.0	57%	1	2	1	2	2	1
**Nic-16**	43	1.6	8%	0	2	1	1	2	1
**Nic-17**	51	-1.0	52%	0	1	1	1	2	1

**Nic-18**	96	-3.0	25%	0	2	1	1	2	1
**Nic-19**	60	-10.6	67%	0	2	1	1	3	1
**Unit**		ml/min/1.73m^{2}/year							

eGFR T0 and kidney biopsy findings compiled from baseline studies (Wijkström et al, Am J Kidney Dis 2013 and Wijkström et al, Am J Kidney Dis 2017).

Abbreviations: T0, baseline; Δ eGFR, change in eGFR; Global scleros, globally sclerosed glomeruli; Segm Sclerosis, segmental sclerosed glomeruli; Interst fibrosis, interstitial fibrosis; Interst inflam, interstitial inflammation; Glom size, glomerular size; Wrinkl GBM, wrinkled glomerular basement membrane; Periglom fibrosis, periglomerular fibrosis.

[a] Percentage of globally sclerosed glomeruli in the biopsy

[b] 0=No segmentally sclerosed glomeruli; 1=presence of segmentally sclerosed glomeruli in the biopsy.

[c] Grading of interstitial fibrosis, interstitial inflammation, and tubular atrophy is defined as 0=no; 1=mild, affecting 25% of the area; 2=moderate, affecting 26-50%; and 3=severe, affecting > 50%.

[d] Grading 0=normal, 1=mild hypertrophy; 2=moderate; and 3=severe hypertrophy.

[e] 0=No; 1=Yes.

[f] Not applicable, not enough tissue for evaluation (less than 5 glomeruli in biopsy).

Table S3: Self-reported data of medication and work exposure during follow-up in the 19 patients with Mesoamerican Nephropathy from Nicaragua.

Pat no	eGFR T0	ΔeGFR per year	ACEi/ ARBs	Allo-purinol	Physical demanding work last 3 months	Agricultural work tasks last 5 years[a]	Exposure to agrochemicals last 3 years?[b]
**Nic-1**	53	-4.2	No	Yes	Yes	Yes	Yes
**Nic-2**	49	1.6	Yes	Yes	Yes	Yes	No
**Nic-3**	50	4.2	Yes	Yes	Yes	Yes	Yes
**Nic-4**	39	2.0	No	No	No	No	No
**Nic-5**	44	-3.4	No	No	No	No	No
**Nic-6**	84	1.4	No	No	No	Yes	No
**Nic-7**	54	-0.2	No	No	No	Yes	Yes
**Nic-8**	56	0.6	No	Yes	No	Yes	No
**Nic-9**	74	-2.4	No	Yes	No	Yes	Yes
**Nic-10**	81	3.2	Yes	Yes	No	No	No

**Nic-11**	79	-1.4	Yes	Yes	Yes	No	Yes
**Nic-12**	33	-2.2	Yes	Yes	No	No	No
**Nic-13**	61	0.4	Yes	Yes	No	No	No
**Nic-14**	42	0.4	Yes	No	No	No	No
**Nic-15**	41	-6.0	Yes	Yes	No	Yes	No
**Nic-16**	43	1.6	No	Yes	Yes	Yes	Yes
**Nic-17**	51	-1.0	No	Yes	No	Yes	No
**Nic-18**	96	-3.0	Yes	Yes	Yes	Yes	Yes
**Nic-19**	60	-10.6	Yes	No	Yes	No	No

Abbreviations: Δ eGFR, change in eGFR; ACEi/ARBs, Angiotensin converting enzyme inhibitors/ Angiotensin II receptor blockers.

[a] Among patients with agricultural work tasks during follow-up, the median duration was 3.0 years (range 0.3-5 years). Only 1 patient worked with sugarcane. Subsistence farming was the most common work task, reported from 6 patients.

[b] Agrochemicals reported were: 2,4D (2,4-Dichlorophenoxyacetic acid) in two patients; Atrazine in two patients; Chlorothalonil in one patient; Chlorpyrifos in one patient; Cypermethrin in four patients; Diphacinone in one patient; Glyphosate in four patients; Paraquat in one patient.
